# Evaluating the Effects of Built Environment on Street Vitality at the City Level: An Empirical Research Based on Spatial Panel Durbin Model

**DOI:** 10.3390/ijerph19031664

**Published:** 2022-01-31

**Authors:** Wanshu Wu, Ziying Ma, Jinhan Guo, Xinyi Niu, Kai Zhao

**Affiliations:** 1 School of Architecture, Huaqiao University, Xiamen 361021, China; maziying0216@163.com (Z.M.); jhguo_work@163.com (J.G.); 2 College of Architecture & Urban Planning, Tongji University, Shanghai 200092, China; niuxinyi@tongji.edu.cn; 3 Key Laboratory of Ecology and Energy-Saving Study of Dense Habitat, Tongji University, Ministry of Education, Shanghai 200092, China; 4 School of Statistics, Huaqiao University, 668 Jimei Rd., Xiamen 361021, China; kzhao_kai@126.com; 5 Modern Applied Statistics and Big Data Research Center, Huaqiao University, Xiamen 361021, China

**Keywords:** street vitality, street activity, built environment, 5D, spatial panel Durbin model, Xiamen Island

## Abstract

There is evidence that the built environment has an influence on street vitality. However, previous studies seldom assess the direct, indirect, and total effect of multiple environmental elements at the city level. In this study, the features of the street vitality on Xiamen Island are described based on the location-based service Big Data. Xiamen Island is the central urban area of Xiamen, one of the national central cities in China. With the help of multi-source data such as street view images, the condition of design that is difficult to effectively measure with traditional data can be better explored in detail on a macro scale. The built environment is measured through a 5D system at the city level, including Density, Diversity, Design, Destination accessibility, and Distance to transit. Spatial panel Durbin models are constructed to analyze the influence of the built environment on the street vitality on weekdays and weekends, and the direct, indirect, and total effects are evaluated. Results indicate that at the city level, the built environment plays a significant role in promoting street vitality. Functional density is not statistically significant. Most of the elements have spatial effects, except for several indicators in the condition of the design. Compared with the conclusions of previous studies, some indicators have different effects on different spatial scales. For instance, on the micro scale, greening can enhance the attractiveness of streets. However, on the macro scale, too much greening brings fewer functions along the street, which inhibits the street vitality. The condition of design has the greatest effect, followed by destination accessibility. The differences in the influences of weekdays and weekends are mainly caused by commuting behaviors. Most of the built environment elements have stronger effects on weekends, indicating that people interact with the environment more easily during this period.

## 1. Introduction

Streets provide places for social exchanges and physical activities. The optimization of the street environment is conducive to promoting daily life and public health. However, auto dependence caused by urban sprawl results in many problems, such as inadequate physical activity, loss of street life, and destruction of street texture, which lead to public health issues and reduce the quality of life [1]. Urban planners have deployed various policies (such as pedestrian-oriented development and compact development) to mitigate auto dependence and to promote street vitality [2].

Street vitality is an important embodiment of urban vitality [3]. It reveals the composition of the relationship between activity and space, which is the first performance index of street space form [4]. Street vitality refers to the people and their activities that can be observed in a specific space and is the product of the number and duration of various activities [5]. All kinds of activities on the street at different times represent vitality [3]. Specifically, street vitality includes not only the number of people staying and crossing the street at different times, the allocation of public facilities, the number of cultural events and celebrations every year, but also the embodiment of vibrant street life and the degree of people’s perception of space [6,7]. Street vitality can be measured by pedestrian flows and activities, the use of public facilities, and the existence of business destinations [7]. Many studies have verified that the built environment, such as functional mix, intensity of development, street facility, scale, and commercial service, can effectively enhance the street vitality [8,9,10,11,12,13,14,15].

There are two major gaps in the literature. First, most studies are conducted on the meso and micro scales, and limited studies assess the influence of the built environment on the street vitality at the city level [15]. Based on the manual survey data or GPS auxiliary equipment, they often focus on one single or several streets, which can hardly reflect the temporal and spatial characteristics of the street vitality across a wide range of time and space [16]. There should be differences in the influencing effects on different geographic scales [14,17,18]. The results on the macro and meso scales have confirmed the differences. For instance, on the macro scale, density has the greatest impact on vitality [19], while excessive population density and over-mixed land use will lead to a reduction in walking behaviors on the micro scale [20]. In addition, the assessment of the built environment relies on the delineation of a geographic region [21,22,23]. The built environment composition of different regions or scales is different [24,25,26]. Therefore, on a larger geographic scale, it is yet to be determined whether the previous arguments are still valid.

Second, previous studies generally distinguish the influencing factors, that is, which can improve street vitality, and which has no influence. Most of them focus on one or several environmental elements, such as density [27,28,29], land use [29,30,31], transportation facility [28,31,32], and design [33,34], to explore the relationship between individual factors and street vitality. Although there are studies measuring the built environment from multiple dimensions, including diversity, density, scale, old buildings, etc., they aim to explore the influencing characteristics of each indicator and to verify the classical theories, without comparing the effects and the magnitudes [15,35]. These assumptions disguise the most effective elements affecting the street vitality, and there is a lack of accurate assessment of the influencing effects, leading to limited guidance in planning practice. Planners may have to prioritize certain built environment dimensions over others because of budget constraints [36]. Once they figure out the effects and contributions, they can design for street vitality.

Therefore, this study attempts to explore the influence of the built environment on the street vitality at the city level and clarify the effects of the environmental elements. The spatial panel Durbin model is used to answer three research questions: (1) Comparing to the micro scale, at the city level, what elements of the built environment have a significant influence on street vitality? (2) Among the built environment elements, which one plays a more important role in affecting street vitality? (3) Are there any changes in the influences of these environmental elements in different time periods?

To answer the above questions, we collected multi-source big data to measure the street vitality and the built environment at the city level, including street networks, location-based service (LBS) data from smartphone applications, street view images, points of interest (POIs), and buildings. With the help of massive samples of LBS big data to perceive the behaviors of street users in a wide range of time and space, the analysis is no longer limited to a single time section and small scales. We can obtain the spatial panel data on the macro scale, thereby mining the temporal and spatial characteristics of the street vitality and the influences. In addition, based on the street view images, the street design features, including the sidewalk, the building space, the greening, and the openness, can be portrayed at the city level, which is difficult to achieve with survey data. That is why the traditional research is mostly carried out on the meso and micro scales.

This study describes the spatiotemporal features of the street vitality at the city level based on LBS big data, measures the built environment through the 5D index system, and analyzes the influence of the built environment on the street vitality by using spatial panel Durbin models. Further, the direct, indirect, and total effects of the built environment elements on weekdays and weekends are evaluated and compared.

This paper is structured and organized as follows. Section 2 provides a literature review. Section 3 introduces the research data and method. The effects of the built environment on the street vitality are explored in Section 4. Section 5 and Section 6 offer discussions and concluding remarks on this study, respectively.

## 2. Related Work

### 2.1. Traditional Theory

Traditional theories can be divided into two categories: activity and environmental design. As far as activities are concerned, the street should provide enough space to stimulate the occurrence of activities. There are more activities and types at the active commercial interface than at the passive commercial interface [5]. The excavation of subtle features is valuable for satisfying the needs of the people on the street. By planning events and activities on the street, more vitality can be stimulated in a specific period [7,37].

In addition, some scholars explore the collective influence of multiple built environment elements on street vitality. Jacobs believes that four conditions should be met to enhance street vitality: functional mix, density, scale, and old building. The four conditions work together to produce vitality [3]. On this basis, Cervero and Kockelman propose the 3D concept of “Density, Diversity and Design” [38]. After that, Cervero et al. introduce “Destination accessibility” and “Distance to transit” to form the 5D concept [39]. Density is the compactness of the built environment, which is highly correlated with residents’ walking willingness and street vitality [36,40]. Diversity is the mixing degree of street functions. Mixed street functions can encourage people to walk and use public transportation and increase the opportunities for people to move on the street [41]. Distance to transit is the convenience to the nearby transit station, which can stimulate more street activities [42]. Destination accessibility reflects the convenience to reach the destination, which can be measured by spatial design network analysis (sDNA) [43]. The 5D framework is often used to assess the street quality and explore the influence of the built environment on street activities [15,35,41,44].

From the perspective of environmental design, coordinated street-side buildings and aspect ratios, clear boundaries, leisurely strolling places, beautiful landscapes, and complete maintenance facilities are indispensable conditions for shaping successful streets [45,46]. All refined details can improve the quality of the street.

### 2.2. Empirical Research

In recent years, as the data environment has become richer and more mature, research on the influence of built environment on street vitality has gradually shifted from qualitative analysis to quantitative analysis. Scholars often select different influencing factors, such as street facility, function, morphology, and accessibility, to construct an index system and further conduct correlation analysis. Previous studies can be sorted out from the following three aspects:

(1) Research on a certain street on the micro scale [15,16,44,47]. Based on pedestrians’ perception of street space, these studies focus on a specific street in the city, evaluating the built environment factors closely related to street design and optimizing street space. They describe the built environment more nuancedly. Perceptions of the built environment are usually assessed through questionnaire surveys. For instance, Sun et al. explored the relationship between walking and the built environment near subway stations in Beijing, and measured perceptions from various aspects such as traffic speed, road width, difficulty of crossing the road, and continuity of sidewalks [32].
(2) Comparative studies of multiple streets. At present, there are relatively a large number of studies on the meso scale. Multiple streets with different forming times [48], location [49], and other attributes [50], or multiple streets with the same attribute [11,14,51] are selected for refined research. According to the characteristics of the research objects, a certain degree of pertinence and diversification is reflected. They are mostly devoted to exploring the built environment factors that affect the walking choices of residents. On the one hand, they focus on a certain type of group, for example, the elderly [20,52] and women [27]. On the other hand, they pay attention to the detailed research of environmental factors and walking behaviors. The built environment is subdivided into perceived characteristics and objective elements, and the connection between them and walking activities is explored [27,29].
(3) Research on the street system on the macro scale. In the early studies, vitality is mainly carried out through field surveys, which is limited by the large cost and difficult to conduct at the city level [35,53]. With the development of information and communication technology, smart sensing devices can be used to obtain urban data with temporal and spatial resolution, and the distribution of a huge number of human activities can be recorded accurately, which provides an important resource for studying vitality [54]. A complete and systematic assessment is constructed in the entire city or in the central urban area [9,10,11,12,13,18,55,56]. Research on the macro scale focuses on multiple dimensions such as land use, traffic accessibility, and employment, identifying the relationship between street vitality and built environment through integrating multi-source data [19,28,33]. Furthermore, some studies determine the spatial autocorrelation and the spatial heterogeneity of the influence of different built environment factors [28,57,58].

Although there are empirical studies that prove the relationships between the built environment and street vitality, there is still no consensus on the specific effects of different environmental factors [25,27,59,60].

First, under the influence of Jacobs’ urban design theory [3], function, density, block size, and building age are all regarded as factors affecting street vitality, which has also been preliminarily confirmed in Seoul [35]. Some scholars believe that the shape of the street (street length, width, aspect ratio, etc.) [61], function (functional density, functional mix, building density, development intensity, etc.) [28,32,33,60,62] and accessibility (bus station density, location, etc.) will affect street vitality [16,29,34]. However, other scholars question the impact of density and accessibility [63,64] and believe that high population density and excessive mixed land use may reduce walking behaviors [20]. Building density and floor area ratio have completely different effects on the vitalities of different development stages [28]. Commercial diversity, bus station density, length, and openness have no obvious correlation with street vitality [16], although some scholars have proved that the density of bus stops is positively correlated with travel behaviors [52,65].

Second, there are differences in the relationships between built environments and walking activities in different spatial regions [25]. Most of the current conclusions are drawn on the meso and micro scales, such as at the neighborhood and the street levels. Whether these factors are still valid on the macro scale is worth verifying. In particular, the results on the macro and meso scales have confirmed differences. For instance, Li et al. find that population and POI density have the greatest impact on the vitality on the macro scale [19], while Cheng holds that excessive population density and over-mixed land use will lead to a reduction in walking behaviors on the micro scale [20]. Sun et al. believe that higher floor area ratio and building coverage attract more people on the micro scale [32], while Lu et al. find that density and floor area ratio have completely different effects on the urban vitalities of different development stages [28]. Further, on the macro scale, people are mobile on the street, thus, vitalities in adjacent streets have mutual influences. The spatial correlation should be introduced when conducting regression analysis, which is usually ignored [57,58].

Third, most studies examine whether the built environment has an impact on the street vitality, but they have not answered the intensity and magnitude of the impacts. They mostly focus on one or several environmental factors, such as density [28,29], land use [29,30,31], transportation facility [28,32], and design [33,34]. Although some studies have measured the built environment from multiple dimensions, they are committed to verifying classical theories, rather than comparing the magnitudes of the effects [15,35]. “Street vitality-built environment” is a complex system. Not all environmental elements directly contribute to the street vitality, and not all environmental elements have the same effect. Alterations in some environmental elements may bring about a large increase or decrease in street activities; however, adjusting other elements may only lead to a slight change. Therefore, the difference in the influence degrees of various environmental factors should be accurately described, which is rarely involved in previous studies and has significance for planning practice.

## 3. Materials and Method

### 3.1. Research Area

Xiamen is one of the national central cities, as well as one of the four earliest special economic zones in China. Further, Xiamen is the leading city in Urban Agglomeration on the West Side of the Straits, enjoying a high reputation for its rapid economic development and coastal landscapes. Due to the livable environment and the typical modern urban space, the built environment here is suitable to conduct research on human settlement and urban development. In addition, Xiamen has undergone the historical evolution of concessions and coastal defense, leading to the formation of a diverse street system. The streets in the historical areas are freely stretched and the interfaces are active. The streets in the living neighborhoods are distributed in a regular and orderly manner, and the streets in the new urban areas have open spaces and broad views, hosting a large number of businesses, offices, and exhibition activities. The street space presents a variety of collage forms and functions, which is an important support to keep streets alive. Thus, the built environment of the streets in Xiamen has complex connotations and external forms, which is a typical sample for exploring the relationship between the built environment and the street vitality.

**Figure 1:**
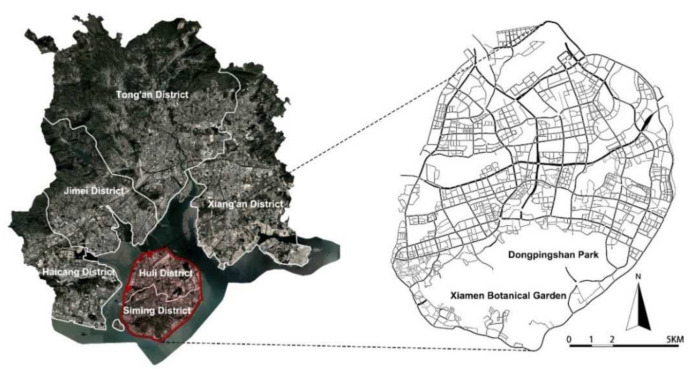
Location and street system on Xiamen Island.

Xiamen Island is the central urban area of Xiamen, including Siming District and Huli District, with a total area of about 132.2 km^2^ (Figure 1). It is the economic and cultural center of the city, carrying most of the living, employment, cultural, and entertainment activities. Xiamen Island has a high density of population and buildings, as well as diverse functions and convenient infrastructures. Separated from other areas by the sea, the functional layout and spatial structure of Xiamen Island are relatively independent. There are differences between Xiamen Island and other districts in terms of function, transportation, population, and development, which should be treated differently. Therefore, Xiamen Island is selected as a typical case for in-depth study.

### 3.2. Research Data

#### 3.2.1. Street Network

As an open-source map data, OpenStreetMap (OSM) has the characteristics of high openness, high timeliness, low acquisition cost, and abundant information. It is widely used in the research of street accessibility [66], land use distribution [67], and street spatial quality [43]. Compared with traditional data, it has more detailed, accurate, and complete information [68]. Therefore, we obtained street network data from OSM. The original data were cleaned, topologically checked, and processed. A total of 2768 streets on Xiamen Island were obtained finally. In addition, referring to the classification in “Xiamen Urban Rail Transit Construction Plan (2011–2020)”, the street system is divided into four levels: expressway, arterial road, secondary trunk road, and branch road.

#### 3.2.2. LBS Big Data

Mobile internet positioning services generate LBS big data actively initiated or passively, recording GPS-accurate locations (up to 10 m) and corresponding time stamps. The precision makes LBS big data particularly suitable for street research. The data are formed by the user’s login, search, sending and receiving information, push, and other events in the mobile phone applications (APPs). Therefore, a trajectory point represents that a mobile phone user is walking or stationary at that location. The LBS big data in this study are the Aurora Mobile anonymous geographic data. Aurora Mobile provides an SDK positioning development environment for smartphone application software and monitors approximately 1.452 million mobile application terminals, including Tencent, Baidu, and other mainstream APPs in China. The data collection mechanism includes periodic updates (a granularity of 1 h/time) and event triggers.

**Figure 2:**
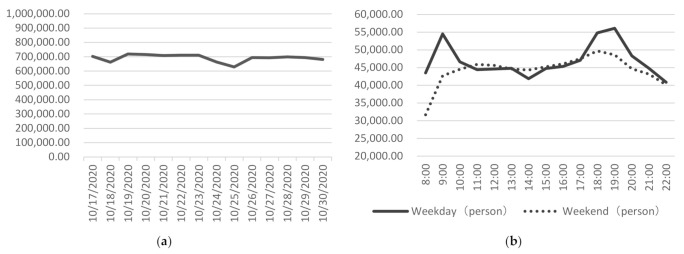
Processing of the LBS big data: (**a**) Changes of street activities during the 14 days; (**b**) changes of street activities during the daytime.

When analyzing the street vitality using LBS data, most of the previous studies collected the data for no more than two weeks [16,31,44,47,69,70]. Through data verification, we find that the data of two weeks is enough to reflect the objective features of the street vitality. Therefore, the data are collected from 17–30 October 2020, including 10 weekdays and 4 weekends, without holidays. During this period, the impact of the COVID-19 in Xiamen is weakened. The temperature is suitable (19.8–26.9 degrees Celsius) and the weather is sunny with no rain. The data processing method is as follows: (1) The LBS big data of all users appearing in Xiamen from 17–30 October 2020 are selected, containing 130,817,778 records and 6.021 million users. The daily data volume is relatively stable (Figure 2a); (2) the location information of the users on each time point during the day (8:00–22:00) is calculated through pushing forward by one hour. A maximum of 329,800 users can be recognized during the weekdays (19:00 on 19 October), and a maximum of 309,200 users during the weekends (18:00 on 17 October), while there are about 2 million permanent residents on Xiamen Island; (3) the users’ locations in the streets on Xiamen Island are selected, and the multi-day averages of 10 workdays and 4 weekends are summarized, respectively (Figure 2b).

#### 3.2.3. Street View Image

**Figure 3:**
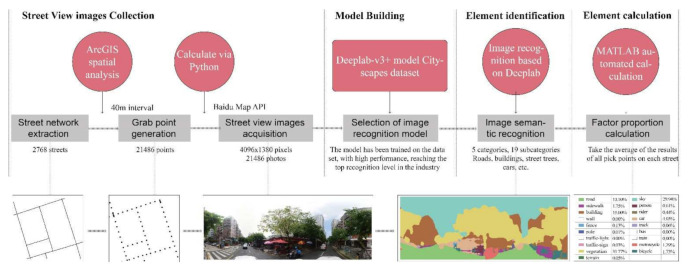
Collection and recognition process of street view images.

Baidu Map is one of the most widely used internet map service providers in China, which is equivalent to Google Maps in Western countries. Baidu Map is also the fastest and most comprehensive platform for street view images in China. Therefore, the street view images in this study were collected through the API interface of the Baidu Map open platform. First, based on the street center lines, the capture points of the street view images were generated at 40 m intervals. For the short street segment, the midpoint of the center line was selected, and a total of 21,486 capture points were obtained. Second, panoramic photos of each capture point were crawled in June 2021. The size of images was set to 4096 × 1380 pixels, and the camera angle covers the 360° omni-directional view of the capture point. The acquired images have unique IDs, as well as latitude and longitude coordinates. A total of 21,486 valid images were obtained. The capture vehicle occupies about one-third in the bottom of the image, which is likely to affect the recognition result. As a result, the bottom one-third of each image is uniformly cut off. Third, the Deeplap-V3+ semantic segmentation tool and Cityscapes Dataset were used to identify the elements in the street view images. The recognition results include 5 categories and 19 subcategories, covering building, road, sky, plant, car, people, and others. Finally, the recognition results were matched with the capture points according to the unique IDs (Figure 3).

#### 3.2.4. POI

The POIs were collected from the 2020 AutoNavi Open Platform. The original data of Xiamen Island contains 169,164 records, with a total of 21 major categories and 236 medium categories. With reference to previous research, POIs were reclassified into 8 categories that are highly related to street vitality: residential (2.86%), corporate (14.21%), government agencies and social groups (1.87%), business (66.37%), education (4.65%), green space (0.33%), transportation (5.23%) and others (4.47%).

#### 3.2.5. Rail Station

Rail stations include metro stations and Bus Rapid Transit (BRT) stations. Using Baidu Map API interface, a total of 53 stations were crawled on Xiamen Island, including 13 stations on Metro Line 1, 20 stations on Metro Line 2, and 20 BRT stations.

#### 3.2.6. Building

The building data were collected from the vector building information on Baidu Map, including the location of the buildings and the number of floors. Based on the QGIS online map and street view images, the building location and floor number were checked and adjusted.

### 3.3. Method

#### 3.3.1. Spatial Unit

**Figure 4:**
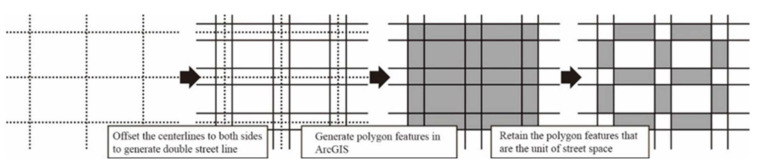
Delineation of street space and division of spatial units.

Street is defined as a compound space that carries residents’ daily life, social activities, and traffic functions [3,8]. The street space is bounded by the architectural interfaces on both sides of the street. The street ranges and spatial units are divided as follows: (1) The center lines are shifted according to the street levels, and the expressway, arterial road, secondary trunk road, and branch road are shifted by 60 m, 40 m, 20 m, and 16 m, respectively, so as to generate two-lane streets; (2) the Google online satellite images are superimposed, and the double lines are calibrated one by one according to the actual street space to ensure that the street space is demarcated by the buildings on both sides of the street; (3) the spatial units are generated by the transformation tool in ArcGIS platform. Divided by the street intersections, a total of 2768 spatial units are obtained (Figure 4).

In terms of spatial experience, there is a complete space between two intersections, and the psychological feelings of the people are continuous. Further, as most of the streets are roadways, intersections produce a certain degree of fragmentation. The built environment has a continuous and direct influence on the activities on the same street segment. Street units between adjacent intersections can undertake all types of activities independently. Therefore, the spatial units are divided by the street intersections.

#### 3.3.2. Variable

##### Dependent Variable

The dependent variable is street vitality. Jacobs believes that street vitality comes from enough people on the street [3]. In order to maintain vitality, the density of active people must be high enough, no matter what purpose these people come here for. In urban areas that can attract many visitors, the number of streets tends to increase. She also emphasizes the importance of time distribution. Vitality refers to people who are active in different periods of time. This time is a short period and is calculated by hour. The number of people on the street is different from the time distribution of them using the street. The number and the time represent different aspects of street vitality. If a street is crowded at a certain time, but it is deserted at other times, it cannot be called a vibrant street.

Gehl, Montgomery, and other scholars conducted further discussions [5,6,7,8]. They hold that street vitality is the external manifestation of the social activities on the street, indicating that the street space is full of various activities based on walking or staying at various times. Therefore, street vitality refers to the density and duration of stationary and slow-moving activities on the street. In recent years, as the data environment has become more abundant, a series of empirical studies have been carried out to define the connotation and measurement of street vitality more clearly. The intensity of the stationary and slow-moving activities is the basic condition for street activity. The denser the activity, the more vibrant the street [11,12,13,15,31,51,71,72]. In addition, the dimension of time is very important but lacks [14,27,31].

Therefore, we define street vitality as the intensity of the stationary and slow-moving activities on the street at various times during the day. The activities here are counted according to the visits of people to the street at different times. Moreover, the people here are not all individuals in the street, but those who are engaged in stationary and slow-moving activities. When people are operating the APPs, they are mostly stationary or slow moving. Therefore, the people located by LBS data on the street can be regarded as static or walking slowly. To clarify the dynamic characteristics of the street vitality and the effects during different periods of time, we use time series to achieve a more detailed measurement (Table 1).

**Figure 5:**
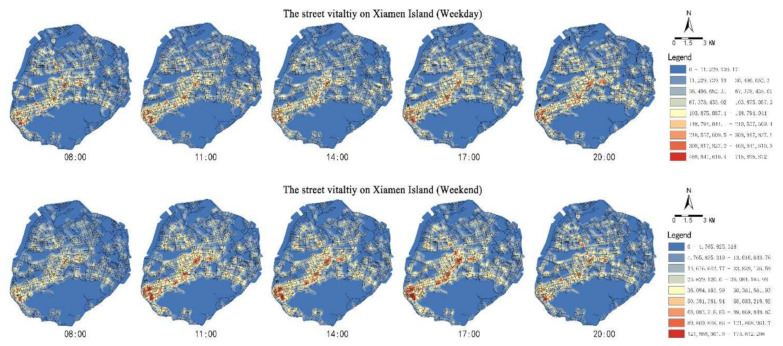
Spatial and temporal distributions of the street vitality on Xiamen Island.

By using LBS big data, the kernel density method is used to visualize the temporal and spatial distributions of the street activities on Xiamen Island (Figure 5). Reasonable bandwidths are selected based on Silverman’s Rule of Thumb (290.33 m on weekdays and 341.09 m on weekends). In general, the streets in the west of Xiamen Island have higher vitality than the east. The vital streets are distributed with dense functions, diverse commerce, and convenient accessibility. On weekdays, the high-value areas changed from a clustered to a striped distribution, reaching the highest value during the evening rush hour. Street vitality changes more smoothly on weekends. The high-value areas are more continuous and wider in the spatial distribution.

##### Independent Variable

The independent variables are the indicators of the built environment. They are the spatial reflections of urban planning and design, and important carriers of street activities. More and more studies have introduced quantitative analysis to better measure the quality of the built environment that was difficult to accurately define in the past, and to provide more refined support for environment optimization [44]. The 5D framework is often used to explore the influence of the built environment on behaviors. We extract key variables which closely relate to street activities. The calculation methods and statistical descriptions of the variables are shown in Table 1. The spatial units of all the indicators are divided by the street intersections. Some indicators are calculated based on the buildings, functions, and traffic facilities in the blocks on both sides of the street; therefore, the statistical area will be buffered to both sides of the street on different scales according to the calculation requirements.

#### 3.3.3. Model Construction

##### Spatial Correlation

To clarify the relationship between the street vitalities, the spatial correlation should be first solved. By using global Moran’s I and Geary’s C, the spatial agglomeration can be described from a global perspective. When conducting the analysis, the first-order geographic adjacency matrix is selected for the following reasons: (1) The topography on Xiamen Island is complex, and there is an amount of non-construction land, including gardens, parks and bays, which break the continuity of space; (2) the streets are connected and people can move freely. The most significant influence on the local vitality is from the adjacent streets that are connected to it, rather than the streets that are close in space but not directly connected.

**Figure 6:**
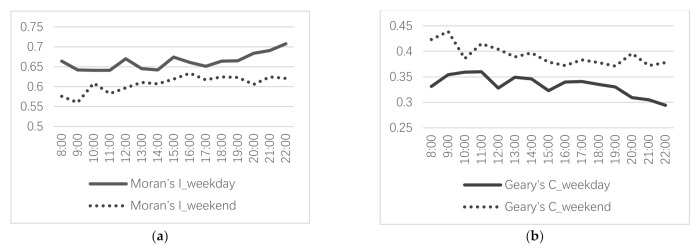
Results of the spatial correlation tests: (**a**) Moran’s I on weekday and weekend; (**b**) Geary’s C on weekday and weekend.

Figure 6a,b, respectively, depict the changes in Moran’s I and Geary’s C over time. Moran’s I is always positive and Geary’s C is always less than 1. Both are significant at the 1% significance level. The results indicate that the street vitality presents a positive spatial autocorrelation and an upward trend over time. The same method is used to test the spatial correlation of the independent variables, and all the variables of the built environment show a positive spatial correlation (Table 2). Therefore, the spatial panel Durbin model is employed, which can assign spatial weights to both the independent variables and the dependent variable.

##### Model Selection

After the collinearity diagnosis, no collinearity phenomenon is found, and the spatial panel Durbin model is introduced to analyze the influence of the built environment on street vitality. The spillover effects of the spatial panel Durbin model are flexible, while those of the spatial lag model (SLM) and the spatial error model (SEM) are not, and those of SEM are even set to zero by construction [73]. Thus, the spatial panel Durbin model is more comprehensive. It can be used as a reference for spatial analysis, and then checking whether it can be degraded to other model forms (such as SLM or SEM) [74]. In addition, the spatial panel Durbin model allows independent variables and control variables to be included in the spatial analysis system at the same time, which can effectively investigate the variables that may have spatial spillovers, thus improving the robustness of the estimation [75].

Therefore, we use the spatial panel Durbin model as a benchmark to determine whether it should be retained or degraded to other models. The results show that the spatial panel Durbin model should be retained for analysis. The model is set as follows:

(1) $$\begin{array}{l} \ln V_{it}^{T}=\rho W\ln V_{it}^{T}+\beta_{1}\ln Density_{it}+\beta_{2}\ln Far\cdots +\beta_{13}\ln tpb_{t}\\ +W\left(\gamma_{1}\ln Density_{it}+\gamma_{2}\ln Far_{it}\cdots +\gamma_{13}\ln tpb_{t}\right)+\mu_{i}+\nu_{t}+\varepsilon_{it} \end{array}$$

where ln indicates that the variable takes the natural logarithm (logarithmic regression can reduce the volatility of the data and possible heteroscedasticity); $V_{it}^{T}$ is the dependent variable of street vitality; i is the street unit; t is the time; T={weekday,weekend} represents weekdays and weekends; ρ refers to the coefficient of spatial autocorrelation, which is used to measure the interdependence of street vitality; $\left\{\beta_{1},\ldots ,\;\beta_{13}\right\}$ is the parameter to be estimated as independent variables; $\left\{\gamma_{1},\ldots ,\;\gamma_{13}\right\}$ is also the parameter to measure the marginal influence of the independent variable on the dependent variable $V_{it}^{T}$ of the neighboring street; $\mu_{i}$ and $\nu_{t}$ represents the fixed effects of street and time, respectively; $\varepsilon_{it}$ is the residual term.

## 4. Results

### 4.1. Overall Influence Characteristics

The spatial panel Durbin models on weekdays and weekends have good interpretability (Table 3). The neighboring street vitalities promote each other. The spatial lag variables represent the spatial spillover effects, indicating that the change of a certain environmental element of the neighboring street leads to the change of the local street vitality.

#### 4.1.1. Density

Intensity of development can directly increase the local street vitality on weekdays and weekends. Improving the intensities of development on both sides of a street can effectively attract people to the local space. In addition, intensity of development shows a reverse inhibitory effect obviously. When the value is higher, the activities are more intensive. There is a competitive inhibitory effect on the neighboring street vitality.

Functional density has no significant influence on the street vitality, and there is no obvious spatial spillover effect either. Compared with the effect of intensity of development, the reason lies in the referentiality of POIs to functions. First, POIs cannot quantify the carrying capacity of the functions. POI is to provide navigation information on the map. A point represents an entity function, which can be a house, a shop, a mailbox, and a bus stop. A low-rise building and a high-rise building, or a snack bar and a large-scale restaurant, may all be a point on the map. However, vitality provided by them for streets is different, and POIs cannot reflect the difference. Second, a POI does not represent only one type of function. For instance, a POI of a restaurant not only represents dining activities but also employment. This complexity cannot be distinguished by POIs. Therefore, this result cannot directly prove that functional density cannot improve street vitality, which needs further demonstration.

#### 4.1.2. Diversity

Functional diversity has a significant effect on the local and neighboring street vitalities. People on the street have more demands for diverse functions; therefore, multiple functions can be fully exerted. The result reflects the correlation between activities and the functional diversity of the street.

#### 4.1.3. Distance to Transit

Metro/BRT stations can enhance street vitality. The closer the distance, the higher the street vitality. It shows a certain negative spatial spillover effect on weekdays, which means that the streets close to the stations are easier to attract people from the surrounding areas, thereby promoting the local street vitality. The local street vitality increases and the neighboring street vitality decreases.

Density of bus stations has a significant effect on improving the local street vitality, and its spatial spillover effect is opposite to the distance to the nearest metro/BRT station. An important reason is the spatial distribution of the two types of traffic stations. The layout of bus stations places more emphasis on walkability and homogeneity within the walking range. Therefore, increasing the bus stations’ density of neighboring streets will also enhance the local street vitality. The metro and BRT stations have a wider service range and generally radiate to non-walkable distances. Therefore, this indicator presents a certain siphon effect.

#### 4.1.4. Design

The influence of street level is significant. The higher the street level, the more vibrant the street is. Meanwhile, it can also increase the vitality of the neighboring street. This result is related to the characteristics of the high-density urban areas in China, especially the old urban areas. The streets around the expressways have dense and complex functions, as well as spacious pedestrian space, which can attract more street activities.

Street length has a significant impact on stimulating the street vitality on weekdays and weekends. Jacobs believes that the shorter the street, the more convenient for people to communicate with each other, thereby promoting the street vitality [3]. However, her conclusions are based on the large western cities, where the street segment is relatively long. The average street length of Xiamen Island is 220 m (Table 1). Especially in the old urban areas around Yundang Lake and Amoy YatSen Road, there are a certain number of extremely short streets less than 7 m. Even if these streets are situated in areas with high urban vitality, the street vitality is still at a low level. This result confirms that the existing theories are mostly based on the background of western cities, and whether it is applicable to other regions needs further verification.

**Figure 7:**
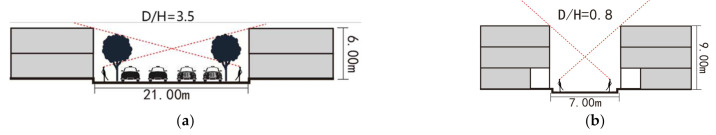
Comparison of streets with different D/H ratios and vitalities: (**a**) The street with high D/H ratio and low vitality (Nanshan Road); (**b**) the street with low D/H ratio and high vitality (Amoy YatSen Road).

D/H ratio has a significant negative effect on street vitality. During the past few decades, the rapid urbanization in China has led to many spacious roads in the city, forming a homogenized and inhuman-scale street space (Figure 7a). In this context, reducing the D/H ratio will help pedestrians feel the architectural style on both sides of streets, creating a cohesive and stable sense of space, thereby effectively defining the walkable space. For instance, some historical streets represented by Amoy YatSen Road are designed with arcades on both sides. The D/H ratio is between 0 and 1 and attracts a large number of people (Figure 7b). This confirms Yoshinobu Abara’s argument that when the D/H is less than 2, it is a more reasonable proportion [45].

Green vision rate has a significant inhibitory effect on the local street vitality, and the space spillover effect on weekends is obvious. This result reflects the difference between macroscopic and microscopic studies. On the micro scale, as far as a single street segment is concerned, a better green environment can provide a comfortable experience, thereby promoting street vitality [16]. However, on the macro scale, this conclusion may not be true. Streets with a higher green vision rate on Xiamen Island are mostly landscape streets. The streets have limited functions and facilities, which make it difficult to maintain a high level of street vitality for a long time. Therefore, in the optimization and renewal of streets, the green environment cannot be improved blindly, and it should be consistent with the street function and people’s demands. Openness reflects a more significant negative influence and the spatial spillover effect is obvious, which is also different from the conclusions on the micro scale. The more open the street, the fewer buildings along the street, leading to less street activity simultaneously. Walkability can improve street vitality. Especially on weekends, as people behave more flexibly and freely on the streets, the spatial effect is significant.

#### 4.1.5. Destination Accessibility

Location has a significant influence. The closer to the city center and the commercial complex, the higher the street vitality. This indicator has a certain “siphon effect” on weekdays. The better the location of a street, the higher the local vitality, while the lower the neighboring street vitality. There is no obvious spatial spillover effect on weekends. People commute on weekdays, as a result, streets with better locations are generally distributed in areas with more jobs, and the spatial effect is more significant.

Two-phase betweenness has a positive effect on the local street vitality and a certain inhibitory effect on the neighboring street vitality. People are more inclined to choose the shortest path with high intelligibility (small angle change) within the line of sight. They can screen out streets with high betweenness based on their preferences, which inhibits the increase of the neighboring street vitality. Therefore, streets with higher betweenness are more attractive to pedestrians.

### 4.2. Direct, Indirect and Total Effects

Although the sign and significance of the lag term’s coefficient in the spatial panel Durbin model are valid, the value does not explain the influence degree of the independent variables on the dependent variable. It is necessary to use statistics such as direct and indirect effects to test the spatial effects [76,77]. According to LeSage and Pace, the effects of multiple independent variables can be further decomposed into direct effects and indirect effects [78]. The direct effect is the influence of a certain variable on the local street vitality. The indirect effect is the influence of a certain variable on the neighboring street vitality; that is, the spatial spillover effect. The total effect refers to the overall influence on the street vitality, including the spatial feedback effect. The total effect is the cyclical process that a certain variable of the local street affects the neighboring vitality, and the neighboring vitality, in turn, affects the local vitality.

Therefore, based on the reasonable verification of the aforementioned models, the direct, indirect, and total effects are used to further explore the influence degree and the contribution of each variable (Table 4).

#### 4.2.1. Direct Effect

In terms of direct effects, the dominant influencing elements on weekdays are ranked as follows according to their contributions: openness, D/H ratio, distance to the nearest metro/BRT station, location, two-phase betweenness, intensity of development, green vision rate, length, functional diversity, street level, density of the bus stations, and walkability. The direct effect of openness is the largest, mainly due to the great contrast between streets with high and low vitalities. Xiamen Island is surrounded by the sea. Island Ring Boulevard occupies nearly three-quarters of the shoreline. Its openness is the highest in the global. However, the street vitality is far below average. Moreover, while the zone from Amoy YatSen Road to Mingfa Plaza has the highest vitality, it has the densest buildings and the lowest openness. Due to the huge difference, openness has a significant direct negative effect on street vitality. The strong direct effect of the D/H ratio reflects the difference between the old and new urban areas. The streets in the old urban areas are narrow with a low D/H ratio but high vitality, represented by Huli District. The new urban areas, represented by those around Wuyuan Bay, have spacious space, a high D/H ratio, and empty streets. The contribution of distance to the nearest metro/BRT station reflects the dependence of commuting on both two modes of transportation.

The dominant environmental elements on weekends are ranked as follows: Openness, D/H ratio, length, two-phase betweenness, location, distance to the nearest metro/BRT station, intensity of development, green vision rate, street level, functional diversity, and walkability. Compared with weekdays, length has replaced the distance to the nearest metro/BRT station as a major contributing element, indicating that residents’ reliance on public transportation is reduced on weekends. In the area with the highest vitality on weekends, the average street length is approximately 180 m. Too long and too short streets are not conducive to gathering activities.

To sum up, the conditions of design and location have the greatest influence on street vitality. Location can create a vigorous environment within a certain range around the street; design is the key to attracting activities, and a perfect street design can always maintain vigor.

#### 4.2.2. Indirect Effect

The indirect effects of the environmental elements are ranked as follows: Openness, functional diversity, street level, location, D/H ratio, density of bus stations, and green vision rate. The spatial spillover effects of design, functional diversity, and distance to transit are the most significant. Design can not only influence the local street vitality but also affect the neighboring vitality. Optimizing the functional configuration and traffic conditions can effectively improve the neighboring street vitalities.

Besides, walkability has no obvious indirect effect on weekdays, and significant indirect effect on weekends. On the contrary, the indirect effect of location on weekdays is significant, and there is no obvious indirect effect on weekends. The result is still related to the restriction of jobs and commuting behaviors.

#### 4.2.3. Total Effect

As far as the total effect is concerned, the most significant element is still design, followed by functional diversity and distance to transit. The total effect of location is not significant. Specifically, the top four elements that have the strongest total effects on weekdays are the same as weekends, including openness, D/H ratio, functional diversity, and street level. In addition, street length, distance to transit, and two-phase betweenness do not show any total effect on weekdays while are significant on weekends.

The influencing conditions on weekdays are more complex, including design, functional diversity, and distance to transit, while design is the most significant contributing condition on weekends. Therefore, the optimization of function and traffic can enhance the street vitality on weekdays and optimizing the street design is always an effective means to maintain vitality.

## 5. Discussion

In this study, the built environment is measured at the city level based on multi-source data. On the meso and micro scales, the information of design can be collected through field investigation, which is difficult to realize on the macro scale. Street view images, POIs, and web-crawled building data provide the possibility to evaluate the detailed features of street design accurately at the city level, including the landscape, building space, sidewalk, and greening. The greening is part of the green infrastructure, emphasizing the connection between green spaces and functions, and contributing to the formation of a green space network [79,80,81,82]. The greening factors on a large scale can be measured more efficiently by using street view images; thus, the street design can be characterized with high quality [44]. On this basis, the 5Ds can be better understood, and the effects of the design indicators can be discussed at the city level.

We used the spatial panel Durbin model to examine the effect and magnitude of the built environment variables on street vitality. Clarifying the most significant elements is conducive to optimizing the street environment in a targeted manner. Based on the conclusions, a phased action strategy can be formulated to maximize the benefits of economic investment under limited resources. For instance, on the macro scale, we should still focus on function and agglomeration. An effective means to maintain full-time vitality is to optimize the space design and to make sure that the greening, the D/H ratio, and the openness are all at a reasonable level.

Comparing street vitalities at different times is to recognize and distinguish the regular commuting behaviors and business travels on weekdays. As this kind of vitality is more purposeful, it may have a weak relationship with the built environment. Residents’ activities are more flexible on weekends, which can better reflect their choice and happiness. Comparing the influence characteristics between weekends and weekdays is helpful to better understand the relationship between street vitality and the built environment. Within a day, street activities also differ at different times. Streets with dense jobs have stronger vitality. In the following research, we will try to clarify the functions of different streets, identify the sources of the street vitality at different times, and distinguish the differences of street vitalities brought by various function combinations, so as to draw more detailed conclusions.

The paper has the following limitations:

First, the research area is Xiamen Island, which is an island surrounded by the sea. Although many scenic streets here are set along the coast, and the design conditions are different from those of plain cities, it will not have a great influence on the accuracy of the results. This influence is highlighted by the increase of coastal streets. Compared with plain cities, street types are more diverse. However, the increase in street types makes the analysis results more detailed and does not affect the rationality of the conclusion.

Second, the built environment indicators of different levels of streets are not quantified in different ranges. When measuring the built environment, we set the index of street level. The influence of street level on street vitality can be reflected in the results. Moreover, we assume that the differences in street levels are not reflected in the ranges, but in the street characteristics. For instance, there may not be much difference between the land uses within 100 m and 150 m around a living street. When calculating the indicators, we set different buffer ranges according to their respective connotations and calculation requirements. These buffer ranges reflect the general feelings of street users and the characteristics of land uses. However, there may be possible limitations of the buffer ranges, and they will be verified and improved after obtaining more accurate data in different research areas.

Third, when calculating openness, green vision rate, and walkability by using street view images, there may be occlusions of mobile objects to the sky, the vegetation, and the pedestrian path. However, this will not cause significant errors. Pedestrians may occlude sidewalks and vegetation, but they are usually too small to cover the sky in panoramic photos. We selected the street view images of the most dynamic streets on Xiamen Island for comparison, and found that the average proportion of “Person” was quite low (less than 0.01). Vehicles may occlude vegetation and pedestrian paths. However, this shelter indicates that the area is not an activity space, but essentially a parking space. As a tourist city, Xiamen keeps its appearance clean and tidy, especially in the central urban area. There are almost no mobile stalls on the street. To sum up, mobile objects will have an occlusion, but it will not cause an obvious error.

Urban residents rely on streets to carry out social activities, business activities, entertainment activities, and to enjoy the outdoor space. Diversified activities have significant differences in spatial-temporal distributions, behavior characteristics, and activity forms. Therefore, there are multi-level requirements for street space and environment, including both material and spiritual demands. Taking street vitality as the guidance to improve the street design and environmental quality can enhance residents’ spatial perception of different types of streets. Effective street design can greatly promote residents’ spatial interactive experience, realize the return of streets to human places, and improve the sense of happiness in the city.

## 6. Conclusions

Based on the LBS big data, we continuously observed the vitalities of almost all the streets on the macro scale for a long period of time and obtained the panel data of street vitality. Further, by using multi-source big data, we quantitatively measured the built environment from the 5D framework at the city level. Considering that both independent variables and the dependent variable have spatial autocorrelation characteristics, the spatial panel Durbin model was introduced to evaluate the direct, indirect, and total effects of the built environment. The differences between weekdays and weekends were further clarified. This study provides new empirical evidence on the impacts of the built environment on street vitality.

The analysis results suggest that at the city level, the built environment plays a significant role in promoting street vitality. Overall, most of the environmental indicators have obvious influences on street vitality, except that functional density is not statistically significant. Moreover, most of the elements have significant spatial effects, except for several indicators in the condition of design, manifesting that the influence of environmental design is more reflected in local streets.

Compared with the conclusions of previous studies, we found that some indicators have different effects on different spatial scales. For instance, on the micro scale, greening can enhance the attractiveness of streets [16]. However, on the macro scale, too much greening brings fewer functions along the street, which inhibits the street vitality. Although design theories hold that short streets can stimulate vitality, the street segment is not as short as possible. According to the empirical results of Xiamen Island, the suitable length is approximately 180 m. Streets that exceed human scales can hardly gather popularity.

Considering the direct, indirect, and total effects, the condition of design has the greatest effect, followed by destination accessibility. At the city level, design is still an effective measure to enhance the street vitality. A high-quality street design can promote the attractiveness of the street and stimulate vitality. In addition, convenient accessibility can bring vitality to the street. Superior locations and perceptive street networks provide more multiple functions and a better space experience for street users, which helps maintain street vitality.

We also clarified the differences in the influences of weekdays and weekends, which are usually caused by commuting behaviors. Street activities on weekdays are mostly dependent on long-distance public transportation. As a result, the influence of metro/BRT station on weekdays is stronger than that on weekends. Most other built environment elements have stronger effects on weekends, indicating that people behave more freely and interact with the environment more easily during this period.

## Figures and Tables

**Table 1:** Index system and statistical description.

Variable	Dimension	Index	Formula	Explanation	Mean	Std	Min	Max
Dependent variable	Street Vitality	Weekday	$\begin{array}{l} V_{t}=\frac{N_{t}}{A_{}}\\ t=8,9,\cdots ,22 \end{array}$	The ratio of the number of street activities in the space unit $N_{t}$ to the area A of the space unit at the t —the time point is used as a measure of street vitality. The LBS big data have a positioning error of 10 m, as a result, when locating the street activities, the space units buffer 10 m on both sides. A is the area of the spatial unit buffering 10 m, and it is a two-dimensional plane area	0.0019	0.0018	0	0.0366
		Weekend			0.0018	0.0018	0	0.0270
Independent variable	Density	Functional density	$\mathrm{Density}=\frac{N}{S}$	Density is the functional density within 55 m around the street, where POI density refers to functional density, *N* is the number of POI facilities within 55 m around the street, and *S* is the area of 55 m around the street.	0.0034	0.0034	0	0.0302
		Intensity of development	$\mathrm{FAR}=\frac{S\_building}{S\_land}$	FAR is the the intensity of development within 100 m of the street, where the plot ratio refers to the the intensity of development, *S_building* is the total construction area within 100 m of the street, and *S_land* is the total land area within 100 m of the street (except street area).	1.9483	1.1412	0	7.7246
	Diversity	Functional diversity	$\mathrm{Diversity}=\sum_{1}^{n}\frac{\mathrm{Pr}*\ln(\mathrm{Pr})}{\ln(n)}$	Diversity is the degree of functional diversity. POI mixing degree is used to refer to the functional diversity. POI is divided into 8 categories, including government agencies, transportation, commerce, education, housing, companies and enterprises, green space and other. Pr is the proportion of the r-th POI within 55 m of the street, and n is the number of POI types within a 55 m range around the street.	0.5276	0.1742	0	0.9526
	Distance to Transit	Distance to the nearest metro/BRT station	Dist_station_i_ = L	Dist_station is the distance between the street and the nearest metro/BRT station, and L is the shortest path distance from the midpoint of the street to the nearest metro station or BRT station around it.	1135.1865	1005.2640	2.6964	7241.9958
		Density of the bus stations	$\mathrm{Den}\_\mathrm{busstation}=\frac{N\_\mathrm{bus}}{S}$	Den_busstation is the density of bus stations within 55 m around the street, *N_bus* is the number of bus stations within 55 m around the street, and *S* is the area of 55 m around the street.	0.0001	0.0002	0	0.0051
	Design	Street level	Level = C	Level is the street level, and C is the road grade of the street. The road grade is divided into four levels: express road, main road, secondary road, and branch road, and assign 1, 2, 3, and 4 in turn.	3.2760	0.8856	1	4
		Length	Length = L_street	Length is the length of the street, and L_street is the length of the center line of the street.	220.2336	200.4348	6.9004	4932.5291
		*D*/*H* ratio	*D* $/H=\frac{D}{H}$	D/H is the aspect ratio of the street, *D* is the average width of the street, and *H* is the average height of the building within 30 m on both sides of the street.	2.2347	2.8794	0.0973	22.6782
		Green vision rate	$\mathrm{Pct}\_\mathrm{green}=\frac{R\_green}{N\_image}$	Pct_green is the green vision rate of the street, *R_green* is the sum of the proportion of green plants in the street view, and *N_image* is the number of street view images crawled by the street.	0.2376	0.1491	0	0.8174
		Openness	$\mathrm{Pct}\_\mathrm{open}=\frac{R\_sky}{N\_image}$	Pct_open is the openness of the street, *R_sky* is the sum of the sky proportions in the street view, and *N_image* is the number of street view images crawled by the street.	0.4025	0.1293	0	0.7036
		Walkability	$\mathrm{Pct}\_\mathrm{sidewalk}=\frac{R\_sidewalk}{N\_image}$	Pct_sidewalk is the walkability of the street, *R_sidewalk* is the sum of the proportions of the sidewalk in the street view, and *N_image* is the number of street view images crawled by the street.	0.0178	0.0154	0	0.1017
	Destination Accessibility	Location	Location = L_centre	Location is the location of the street, and L_centre is the shortest path distance from the center of the street to the nearest city-level center, district-level center, and commercial complex.	1888.8249	1205.6027	3.6662	7825.3683
		Two phase betweenness	${\mathrm{TPB}}_{t}(x)=\sum_{y\in N}\sum_{z\in Ry}OD\left(\mathrm{y,z,x}\right)$	TPBt(x) is the betweenness of the street, *OD*(y,z,x) is the number of nodes passing through node x within the search radius *R*, and Links(y) is the total number of nodes within the search radius *R* for each node y, P(z) is the weight of node z, *R* = 1000 m.	3.9326	3.3214	0.3333	29.0884

**Table 2:** Spatial correlation of independent variables.

System	Indicator	Moran’s I		Geary’s C	
		Coefficient	*p*-Value	Coefficient	*p*-Value
Density	Functional Density	0.244 *** (17.374)	0.000	0.759 *** (−16.086)	0.000
	Construction Strength	0.771 *** (55.449)	0.000	0.233 *** (−34.358)	0.000
Diversity	Mixed Function	0.459 *** (32.845)	0.000	0.526 *** (−24.442)	0.000
Distance to Transit	Distance to the nearest subway/BRT station	0.868 *** (61.691)	0.000	0.138 *** (−58.690)	0.000
	Bus stations density	0.050 *** (3.571)	0.000	0.961 *** (−2.713)	0.003
Design	Street level	0.287 *** (20.381)	0.000	0.710 *** (−20.276)	0.000
	Street length	0.344 *** (24.444)	0.000	0.649 *** (−24.330)	0.000
	Street D/H	0.498 *** (35.376)	0.000	0.494 *** (−35.132)	0.000
	Green View Index	0.634 *** (45.151)	0.000	0.366 *** (−39.459)	0.000
	Sky-openness Index	0.314 *** (22.577)	0.000	0.679 *** (−15.530)	0.000
	Walkability	0.228 *** (16.234)	0.000	0.765 *** (−15.992)	0.000
Destination Accessibility	Location	0.933 *** (66.380)	0.000	0.069 *** (−61.945)	0.000
	Two Phase Betweenness	0.330 *** (23.436)	0.000	0.661 *** (−23.620)	0.000

*** is significant at the 1% significance level. Z-values are in brackets.

**Table 3:** Estimated results of the spatial panel Durbin models.

Time		Weekday		Weekend	
Variable		Independent Variable	Spatial Lag Variable	Independent Variable	Spatial Lag Variable
Spatial autocorrelation coefficient $\left(\rho \right)$		0.457 *** (92.22)	——	0.265 *** (45.45)	——
Density	Functional Density	0.012 (1.17)	−0.001 (−0.08)	0.010 (0.73)	0.019 (0.90)
	Intensity of development	0.170 *** (7.50)	−0.077 *** (−2.85)	0.157 *** (5.12)	−0.067 * (−1.84)
Diversity	Functional diversity	0.054 *** (4.79)	0.112 *** (6.66)	0.065 *** (4.25)	0.189 *** (8.28)
Distance to Transit	Distance to the nearest metro/BRT station	−0.229 *** (−4.87)	0.119 ** (2.31)	−0.188 *** (−2.96)	−0.006 (−0.09)
	Density of the bus stations	0.048 *** (6.61)	0.044 *** (3.31)	0.051 *** (5.16)	0.071 *** (3.96)
Design	Street level	−0.047 ** (−1.98)	−0.078 ** (−2.29)	−0.077 ** (−2.40)	−0.068 * (−1.84)
	Length	0.084 *** (3.11)	−0.079 * (−1.77)	0.237 *** (6.52)	−0.017 (−0.28)
	D/H ratio	−0.239 *** (−8.98)	0.033 (0.85)	−0.261 *** (−7.23)	−0.049 (−0.92)
	Green vision rate	−0.080 *** (−4.18)	−0.029 (−1.22)	−0.123 *** (−4.77)	−0.111 *** (−3.46)
	Openness	−0.208 *** (−6.18)	−0.320 *** (−5.89)	−0.310 *** (−6.81)	−0.689 *** (−9.33)
	Walkability	0.031 ** (2.33)	0.003 (0.16)	0.036 ** (2.01)	0.083 *** (3.00)
Destination Accessibility	Location	−0.209 *** (−2.64)	0.194 ** (2.32)	−0.227 ** (−2.11)	0.178 (1.56)
	Two phase betweenness	0.179 *** (6.76)	−0.118 *** (−2.72)	0.226 *** (6.30)	−0.130 ** (−2.21)
$\sigma^{2}$		0.188 *** (136.75)		0.531 *** (138.67)	
Log-likelihood		−3.058 × 10^4^		−5.006 × 10^4^	
R^2^		0.567		0.508	
Number of samples		41,475			

***, **, and * are significant at the 1%, 5%, and 10% significance levels, respectively. Z-values are in brackets.

**Table 4:** Decomposition results of the influence of the built environment on the street vitality.

Dimension	Variable	Weekday			Weekend		
		Direct Effect	Indirect Effect	Total Effect	Direct Effect	Indirect Effect	Total Effect
Density	Functional Density	0.012 (1.25)	0.006 (0.24)	0.018 (0.63)	0.011 (0.90)	0.028 (1.03)	0.039 (1.34)
	Intensity of development	0.169 *** (8.00)	0.002 (0.05)	0.171 *** (5.95)	0.154 *** (5.28)	−0.032 (−0.84)	0.122 *** (4.23)
Diversity	Functional diversity	0.074 *** (7.07)	0.231 *** (9.14)	0.305 *** (11.66)	0.081 *** (5.66)	0.264 *** (9.65)	0.345 *** (13.15)
Distance to Transit	Distance to the nearest metro/BRT station	−0.229 *** (−5.49)	0.027 (0.48)	−0.202 *** (−5.36)	−0.195 *** (−3.37)	−0.068 (−0.98)	−0.263 *** (−6.97)
	Density of the bus stations	0.059 *** (7.66)	0.115 *** (4.70)	0.174 *** (6.02)	0.059 *** (6.02)	0.112 *** (4.52)	0.171 *** (5.89)
Design	Street level	−0.061 ** (−2.48)	−0.169 *** (−3.19)	−0.230 *** (−3.73)	−0.090 *** (−2.78)	−0.240 *** (−4.33)	−0.330 *** (−5.36)
	Length	0.078 *** (2.78)	−0.071 (−0.92)	0.007 (0.08)	0.242 *** (6.68)	0.058 (0.73)	0.300 *** (3.27)
	D/H ratio	−0.250 *** (−9.59)	−0.131 ** (−2.23)	−0.382 *** (−6.16)	−0.270 *** (−7.64)	−0.154 ** (−2.41)	−0.424 *** (−6.82)
	Green vision rate	−0.091 *** (−5.14)	−0.110 *** (−3.68)	−0.201 *** (−6.73)	−0.135 *** (−5.63)	−0.184 *** (−5.41)	−0.319 *** (−10.69)
	Openness	−0.268 *** (−7.76)	−0.705 *** (−8.36)	−0.973 *** (−10.17)	−0.368 *** (−8.04)	−0.991 *** (−11.25)	−1.359 *** (−14.20)
	Walkability	0.033 *** (2.62)	0.029 (0.88)	0.062 * (1.71)	0.042 ** (2.53)	0.118 *** (3.41)	0.160 *** (4.43)
Destination Accessibility	Location	−0.192 ** (−2.57)	0.162 * (1.83)	−0.030 (−0.67)	−0.215 ** (−2.06)	0.145 (1.25)	−0.070 (−1.56)
	Two phase betweenness	0.176 *** (6.72)	−0.063 (−0.94)	0.113 (1.50)	0.224 *** (6.45)	−0.093 (−1.31)	0.131 * (1.74)

***, **, and * are significant at the 1%, 5%, and 10% significance levels, respectively. Z-value is in brackets.

## Data Availability

Not applicable.
